# Mutation of *ZmDIR5* Reduces Maize Tolerance to Waterlogging, Salinity, and Drought

**DOI:** 10.3390/plants14050785

**Published:** 2025-03-04

**Authors:** Zhixiong Zhao, Tao Qin, Hongjian Zheng, Yuan Guan, Wei Gu, Hui Wang, Diansi Yu, Jingtao Qu, Jihui Wei, Wen Xu

**Affiliations:** 1 Shanghai Key Laboratory of Agricultural Genetics and Breeding, Shanghai Engineering Research Center of Specialty Maize, Crop Breeding and Cultivation Research Institution/CIMMYT-China Specialty Maize Research Center, Shanghai Academy of Agricultural Sciences, Shanghai 201403, China; 2 College of Agriculture, Xinjiang Agricultural University, Ürümqi 830052, China

**Keywords:** *DIRs*, *ZmDIR5*, drought stress, salt tolerance, waterlogging tolerance

## Abstract

The *DIR* (Dirigent) gene family plays a multifaceted role in plant growth, development, and stress responses, making it one of the key gene families for plant adaptation to environmental changes. However, research on *ZmDIRs* in maize remains limited. In this study, we identified a member of the maize *DIR* gene family, *ZmDIR5*, whose promoter region contains numerous elements associated with responses to abiotic stresses. *ZmDIR5* is upregulated in response to waterlogging, salt, and drought stresses, and its protein is localized in the endoplasmic reticulum. Subsequent studies revealed that ZmDIR5-EMS (ethyl methane sulfonate) mutant lines exhibited reduced growth compared to WT (wild-type) plants under waterlogging, salt, and drought stress conditions. The mutant lines also demonstrated a relatively higher accumulation of malondialdehyde and reactive oxygen species, lower synthesis of proline and total lignans, and decreased antioxidant enzyme activity under these stress conditions. Additionally, the mutant lines displayed impaired sodium and potassium ion transport capabilities, reduced synthesis of abscisic acid and zeatin, and decreased expression of related genes. The mutation of *ZmDIR5* also inhibited the phenylpropanoid biosynthesis pathway in maize. These results indicate that *ZmDIR5* serves as a positive regulator of maize tolerance to waterlogging, salt, and drought stresses.

## 1. Introduction

Maize (*Zea mays* L.) is a globally cultivated cereal crop and one of the world’s most important food sources, providing a substantial amount of energy and nutrition for humans, while also serving as feed and industrial raw material in the livestock and bioenergy sectors [1]. As sessile organisms, plants cannot move actively, which necessitates the development of complex physiological and genetic mechanisms to adapt to their ever-changing environments [2]. With the intensification of global climate change, future environmental changes are anticipated to be more severe, posing significant challenges to global ecosystems and agricultural production [3]. Throughout its growth and development, maize may encounter various environmental stresses, including drought, salt stress, and waterlogging. These stresses can adversely impact the physiological and biochemical processes of maize, leading to growth inhibition, reduced yield, and even mortality. Therefore, accelerating in-depth research on maize’s stress adaptation mechanisms, as well as the exploration and utilization of stress-tolerant gene resources, is of great significance for enhancing maize yield and stability.

With the rapid advancement of genomics, transcriptomics, and metabolomics, an increasing number of plant stress-related metabolic genes have been identified, among which *DIRs* represent a significant gene family. *DIR* genes encode a class of Dirigent proteins that play crucial roles in plant growth, development, and stress responses [4]. Recent studies have elucidated the functions of *DIR* genes in stress responses across various plant species. For example, *CaDIR7* in pepper has been shown to be involved in defense against both biotic and abiotic stresses; silencing *CaDIR7* results in reduced root vigor, thereby increasing sensitivity to *P. capsici* and salt stress [5]. In wheat, the loss of function of *TaDIR-B1* has been associated with increased resistance to Fusarium crown rot (FCR) [6]. In sugarcane, *ScDIR7*/*DIR9*/*DIR11*/*DIR40* enhance drought tolerance in transgenic tobacco by boosting its antioxidant capacity [7]. Overexpression of *PtDIR11* in poplar significantly increases the total lignan content within the plant and influences multiple pathways, including phenylpropanoid biosynthesis, to enhance disease resistance [8]. Additionally, overexpression of *VvDIR4* in Arabidopsis and grape can modulate hormone signaling pathways to improve resistance to anthracnose [9]. In rice, the *OsDIR55* gene enhances salt tolerance by modifying the root diffusion barrier [10]. In the expression profiling analysis of the DIR gene family in *Medicago truncatula*, 29 *MtDIRs* were subjected to abiotic stress treatments. It was observed that different gene members exhibited high expression levels under drought, cold, and salinity stresses [11]. Researchers identified 37 *VrDIRs* in mung bean (*Vigna radiata*), and expression profiling revealed that *VrDIR* genes displayed diverse responses to various stress conditions, with some genes specifically responding to either drought or salt stress [12]. In other legumes, such as pigeon pea (*Cajanus cajan*), 25 *CcDIRs* were identified, among which *CcDIR2* and *CcDIR9* responded specifically to salt stress [13]. In potato (*Solanum tuberosum*), 31 *StDIRs* were identified, and these genes demonstrated specific responses to cold stress, salt stress, ABA, and drought stress, thereby providing new candidate genes for enhancing potato resistance to environmental stresses [14]. However, research on *DIR* genes in maize remains limited, particularly concerning their responses to drought and waterlogging stresses.

Currently, the identification of stress-tolerant genes in maize has progressed rapidly, leading to the discovery of numerous related genes. However, the majority of these genes respond exclusively to a single type of stress. Research on maize *DIR* genes remains relatively limited, particularly concerning their roles in significant abiotic stresses such as drought. In this study, we identified the gene *ZmDIR5*, which is significantly upregulated under waterlogging stress. By constructing ZmDIR5-EMS mutant lines, we systematically investigated the functional performance of this gene under various stress conditions, including waterlogging, salt, and drought. Through the design of multiple stress environments, we preliminarily elucidated the function of *ZmDIR5* from several perspectives, including gene expression regulation, metabolic synthesis, antioxidant defense, and hormone synthesis, with the aim of identifying a novel stress-tolerant gene in maize capable of responding to multiple abiotic stresses. This study employed a variety of experimental approaches to comprehensively explore the role of *ZmDIR5* in maize stress tolerance. The results demonstrated that *ZmDIR5* significantly enhances maize tolerance to waterlogging, salt, and drought stresses. These findings not only provide new insights into the molecular mechanisms of *ZmDIR5* in maize stress tolerance but also offer valuable genetic resources for future stress-tolerant maize breeding. Further research on the function and regulatory network of *ZmDIR5* holds promise for developing new maize varieties with enhanced adaptability to adverse environments, thereby improving the stability and sustainability of agricultural production.

## 2. Materials and Methods

### 2.1. Maize Materials and Stress Treatment Methods

The maize materials utilized in this study included the B73 inbred line, along with homozygous mutant lines of *ZmDIR5* in the B73 background derived from EMS mutagenesis. The EMS mutants were obtained from the maize EMS mutant library (http://maizeems.qlnu.edu.cn/ (accessed on 6 June 2021)). The website provides detailed information on the acquisition process and applications of EMS mutant maize seeds. Uniformly sized seeds were selected and disinfected with sodium hypochlorite before being placed in a dark treatment at 26 °C to promote germination. Following germination, the seedlings were transferred to 10 cm^3^ black square pots filled with a substrate mixture of peat soil, vermiculite, and perlite in a 1:1:1 ratio. The seedlings were grown in a controlled environment chamber with a 26/22 °C day/night temperature, a 14/10 h light/dark cycle, 60% relative humidity, and daily equal watering. Upon reaching the three-leaf stage, the seedlings were divided into four groups (A, B, C, and D) for distinct treatments. Group A served as the control and received standard treatment; Group B was subjected to waterlogging, where water was maintained at approximately three centimeters above the aboveground portions of the maize seedlings through daily top-ups; Group C received salt treatment via irrigation with a 200 mM NaCl solution; and Group D underwent drought treatment using a 20% PEG6000 (polyethylene glycol) solution for irrigation. The irrigation solution for each group was prepared with Hoagland nutrient solution. The waterlogging treatment lasted for 30 days, while the salt and drought treatments were administered for 16 days. At various time points, the second vegetative leaf of the maize seedlings was harvested, rapidly frozen in liquid nitrogen, and stored at −80 °C for subsequent analysis. Additionally, a group of WT maize was prepared for natural drought experiments. Watering was stopped at the three-leaf stage and the drought was maintained for 16 days. The second leaf was regularly collected to detect the dynamic changes in *ZmDIR5* gene expression under natural drought conditions. Natural drought treatment requires a long duration and cannot maintain a stable stress environment. Therefore, in the experiment, natural drought treatment was only used for an induction expression analysis, while PEG treatment was used to simulate drought stress conditions for the remaining experiments. For tissue expression pattern analysis, WT materials normally cultivated to the flowering stage (67 d) in a greenhouse were used, and roots, stems, leaves, silks, and pollen tissues were collected for quantitative analysis of *ZmDIR5* expression levels.

### 2.2. Tobacco Materials and Subcellular Localization Analysis

The tobacco materials utilized in this study were *Nicotiana benthamiana*, sourced from our laboratory. Tobacco plants were cultivated in a light incubator at 26 °C with a light/dark cycle of 14/10 h, using 10 cm^3^ black square pots filled with nutrient-rich soil as the planting medium. After four weeks of growth, these tobacco plants were employed for subcellular colocalization experiments. The *ZmDIR5* sequence was amplified, cloned, and subsequently inserted into the vector pCAMBIA1300-GFP. Control protein genes, *AtH2B* and *AtWAK-HDEL*, were incorporated into the vector pCAMBIA1300-RFP. The constructed vectors were utilized for plasmid extraction, which was then transformed into *Agrobacterium tumefaciens* strain GV3101. A mixed solution was prepared and injected into the leaves of four-week-old tobacco plants, which were then kept in the dark for two days. The marked tobacco leaves, injected with *Agrobacterium*, were employed to prepare slides for observation and photography using a laser confocal microscope [15]. The localization control protein AtH2B is a histone structure situated in the nucleus and is a component of the nucleosome [16]. The other localization control protein, AtWAK, belongs to a class of cell wall-associated kinases (WAKs) associated with the endoplasmic reticulum (ER) [17]. HDEL serves as an ER retention signal peptide, typically located at the C-terminus of proteins, facilitating the localization or retention of proteins within the ER [18]. The AtWAK-HDEL protein is specifically localized in the endoplasmic reticulum.

### 2.3. Establishment of ZmDIR5-EMS Mutant Lines

The first-generation seeds of *ZmDIR5* mutants were obtained from the maize EMS mutant library. The construction of the EMS mutant library aims to facilitate functional gene mining and genomic research in maize. Seeds of the maize inbred line B73 were treated with 0.5% EMS in phosphate buffer (pH 7.0) for 8 h at 25 °C with gentle shaking. Following treatment, the seeds were thoroughly rinsed with distilled water for 2 h to eliminate residual EMS before being sown in the field. The first generation of mutant plants was self-pollinated to produce the second generation of seeds, which were sequenced at the three-leaf stage to confirm mutations in the target genes for further analysis [19]. We obtained the first generation of mutant materials from the EMS mutant library, confirmed by sequencing to carry mutations in *ZmDIR5*, which served as experimental materials. Subsequently, we obtained background-purified *ZmDIR5* mutants through three consecutive rounds of backcrossing, followed by two generations of selfing. In each generation, seeds were subjected to PCR amplification using designed primers, and sequencing was performed to compare and identify homozygous materials carrying *ZmDIR5* mutations. These homozygous materials were utilized for propagation and stress experiments. WT specimens derived from the selfing of *ZmDIR5* heterozygous mutants served as the control group in these experiments. This study utilized two mutant lines: EMS4-03f631 (M1) and EMS4-1ee828 (M2), with detailed information provided in Supplementary Table S1. The sequence alignment of the *ZmDIR5* gene and protein between the mutants and the wild type is illustrated in Figure S1. The mutants were backcrossed and self-crossed multiple times to achieve homozygosity. DNA was extracted from the leaves of each maize generation, and positive mutation sites were identified using PCR and agarose gel electrophoresis, with primers listed in Supplementary Table S2. Homozygous lines of each mutant, along with the B73 wild type, were selected for stress treatment experiments.

### 2.4. Bioinformatics Analysis Methods

The *ZmDIR5* gene was identified through a reanalysis of a transcriptomic database (NCBI SRA archive accession number is PRJNA687609) concerning waterlogging stress in maize, originally shared by Yao et al. (2021) [20]. We downloaded and reanalyzed this database, identifying the upregulated gene *Zm00001d006873*, which we designated as *ZmDIR5* for further exploration. To validate the expression pattern of this gene, we subjected B73 maize seedling materials to waterlogging treatment, extracted RNA, and conducted RT-qPCR. The results confirmed that the expression pattern of *ZmDIR5* was consistent with that observed in the transcriptomic database. The gene and protein information for *ZmDIR5* were obtained from the MaizeGDB and NCBI databases. The protein structure of *ZmDIR5* was predicted using the SWISS-MODEL online platform (https://swissmodel.expasy.org/). The domain diagram of the *ZmDIR5* protein was created using the IBS 2.0 online tool (https://www.ibs.renlab.org/#/home (accessed on 6 March 2023)). The sequence located 2000 bp upstream of the *ZmDIR5* start codon was retrieved, and promoter element analysis of the *ZmDIR5* promoter region was conducted using the PlantCare online resource (http://bioinformatics.psb.ugent.be/webtools/plantcare/html/ (accessed on 15 March 2023)). Visualization predictions were performed using Tbtools software (version 2.0). Amino acid sequences of DIR proteins from rice, wheat, and Arabidopsis were also retrieved from the NCBI database. Multiple-sequence alignment analysis was carried out using DNAMAN software (version 9.0), and a phylogenetic tree was generated using MEGA-X 5.0 based on the neighbor-joining method.

### 2.5. Measurement of Growth Indices

Ten seedlings were randomly selected from each group. The height of the aboveground portion and root length were measured using a ruler, while fresh and dry weights were determined using an analytical balance. Following the measurement of fresh weight, the samples were dried in an oven at 70 °C for two days until a constant weight was achieved, after which the dry weight was measured. Chlorophyll content was assessed using a kit (Product Number R30054) from Yuanye Biotech (Shanghai, China). The method involved extracting chlorophyll with an organic solvent according to the manufacturer’s instructions. The absorbance (OD) was measured using a microplate reader, and chlorophyll content was calculated using the formula: total chlorophyll content (mg/g) = (20.2A645 nm + 8.02A663 nm) × V/(1000 × W), where A663 nm and A645 nm represent the absorbance at 663 nm and 645 nm, respectively; V is the volume of the extraction solution used for different treatments, in milliliters; and W is the fresh weight of the maize leaf, in grams. Chlorophyll content was measured in three biological replicates.

### 2.6. Measurement of Physiological Indices and Antioxidant Enzyme Activity

The determination of proline (Pro), malondialdehyde (MDA), hydrogen peroxide (H_2_O_2_), and superoxide anion (O^2−^) content, as well as the activities of superoxide dismutase (SOD), catalase (CAT), and peroxidase (POD) enzymes, along with root activity, were conducted using corresponding kits obtained from Solarbio Science & Technology (Beijing, China). Following the manufacturer’s instructions, a microplate reader was employed to measure the final optical density (OD) values, which were subsequently converted into concentrations using the fixed formulas provided in the manual. The Pro content was calculated based on a standard curve equation (y = 0.0291x − 0.0039, R^2^ = 0.9991). Each index for every sample group was measured in triplicate.

### 2.7. Measurement of Na^+^ and K^+^ Content

Fresh second leaves of maize were harvested, dried in an oven at 75 °C until a constant weight was achieved, and then ground into a fine powder. Approximately 0.1 g of each sample was placed into a 15 mL centrifuge tube. Following the preparation of all samples, 10 mL of 100 mmol/L acetic acid was added to each tube. The tubes were subsequently placed in a 90 °C water bath for 2 h, with gentle shaking occurring every 10 min. After removal from the water bath, the samples were allowed to cool to room temperature before being filtered through filter paper into a 25 mL volumetric flask, which was then filled to the mark with deionized water. Standard solutions of Na^+^ and K^+^ at concentrations of 1, 5, 10, 50, and 100 μg/mL were prepared, and the concentrations of Na^+^ and K^+^ were determined using a flame photometer [21]. Each index for each sample group was measured in triplicate.

### 2.8. Measurement of Total Lignan Content

Methanol was utilized as the extraction solvent for total lignans. Fresh maize leaves were ground into a powder and combined with methanol at a ratio of 1 g to 20 mL. The resulting mixture was subjected to ultrasonic extraction, set at a power of 2500 W and a temperature of 40 °C for a duration of 1 h. Following centrifugation, the supernatant was collected and analyzed using the Plant Lignan ELISA Kit (Enzyme-linked Biotech, Shanghai, China). The absorbance at 450 nm was measured with a microplate reader, and the lignan content was calculated using the standard curve regression equation (y = 0.235x + 0.0239, R^2^ = 0.996). Each sample group was assessed in triplicate.

### 2.9. Measurement of ABA and Zeatin Content

The content of abscisic acid (ABA) and zeatin was determined by Personal Biotech (Shanghai, China). ABA was measured using liquid chromatography–mass spectrometry (LC–MS) [22], while zeatin was quantified using gas chromatography–mass spectrometry (GC–MS) [23]. Each sample group consisted of six biological replicates.

### 2.10. Total RNA Extraction and Reverse Transcription

Total RNA was extracted from various parts of maize using the Tiangen TRIZol Total RNA Extraction Kit (Tiangen, Beijing, China) and subsequently treated with DNase to eliminate any genomic DNA contamination. Following this, the total RNA was reverse transcribed into cDNA templates using the HiScript III All-in-one RT SuperMix Kit (Vazyme, Nanjing, China), in accordance with the manufacturer’s instructions.

### 2.11. Quantitative Real-Time PCR

RT-qPCR was conducted using the ChamQ SYBR Color qPCR Master Mix Kit (Vazyme, Nanjing, China). Primers necessary for the experiment were designed utilizing Primer 5.0 software (see Supplementary Table S2). All primers were synthesized by Tsingke Biotech (Shanghai, China). Quantitative PCR was performed on an Applied Biosystems QuantStudio™ 6 Flex Real-Time PCR System from Thermo Fisher Scientific, with *ZmACTIN1* serving as the reference gene. The reaction conditions and system were established according to the manufacturer’s instructions. Each sample included three biological replicates and three technical replicates, with the Ct values among replicates differing by less than 0.5. The relative expression levels of genes were calculated using the 2^−ΔΔCT^ method [24].

### 2.12. Statistical Analysis

All experiments in this study were conducted at least three times, and the results from representative datasets are presented. Statistical analyses were performed using GraphPad Prism (version 9.3.0). The statistical evaluations employed one-way analysis of variance (ANOVA) with multiple comparisons, followed by Tukey’s tests. Results were deemed statistically significant at *p* < 0.05.

## 3. Results

### 3.1. Identification and Bioinformatics Analysis of ZmDIR5

**Figure 1:**
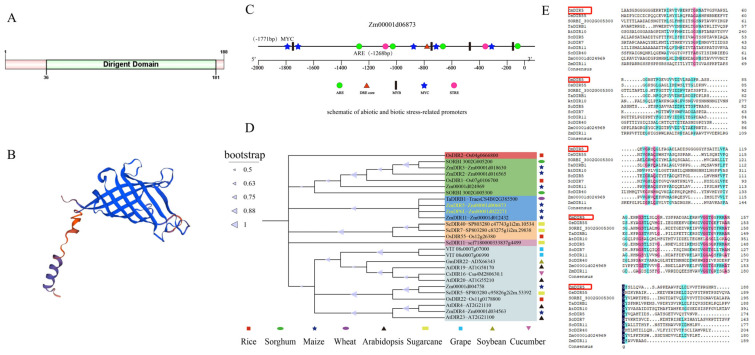
(**A**) Protein domain structure of ZmDIR5. (**B**) Predicted protein model of ZmDIR5; the protein structure contains only two types of secondary structures, with blue representing β-sheets and red representing α-helices. (**C**) Analysis of stress-related promoter elements in the promoter region of ZmDIR5, with distinct shapes representing various promoter elements: ARE (antioxidant response element); MYB (MYB transcription factor binding site); MYC (MYC transcription factor binding site); STRE (stress response element); DRE-core (dehydration-responsive element core). (**D**) Phylogenetic tree analysis of ZmDIR5, indicating different crops with unique shapes, where different colors represent different subfamilies. (**E**) Multiple-sequence alignment of ZmDIR5 with DIR proteins from other crop species, the ZmDIR5 is indicated by the red box.

Through the analysis of transcriptomic data from maize under waterlogging stress (NCBI SRA archive accession number is PRJNA687609) [20], we identified a significantly upregulated gene, *Zm00001d006873*, among the differentially expressed *ZmDIRs*. We obtained gene information from the MaizeGDB and NCBI databases, amplified, and re-sequenced the gene to verify its identity. The gene was determined to be 567 bp in length, containing a single exon and encoding 188 amino acids. A CD search revealed that the protein structure domain comprises only one Dirigent domain (Figure 1A), which aligns with the classical characteristics of *DIR* genes and Dirigent proteins [25]; thus, it was designated as *ZmDIR5*. We utilized SWISS-MODEL to construct a protein structure model of *ZmDIR5*, which exhibited structural features similar to the Dirigent protein model (PF03018) in the Pfam database (Figure 1B). Further analysis of the promoter elements located 2000 bp upstream of the *ZmDIR5* start codon, using the PlantCare online tool, revealed the presence of five key stress-related promoter elements: MYB, MYC, ARE, STRE, and DRE-core [26] (Figure 1C). The MYB, MYC, and STRE elements are responsive to drought and salt stresses, the DRE-core element responds to drought stress, and the ARE element is associated with oxidative stress. To further elucidate the evolutionary relationships and predict the function of *ZmDIR5,* we queried the NCBI database for *DIR* genes from nine species, including rice, wheat, and Arabidopsis, totaling 27 genes, and constructed a phylogenetic tree. Our analysis revealed that *ZmDIR5* exhibits high homology with genes such as *ZmDIR11*, *TaDIRB1*, *ScDIR40*, and *OsDIR55* (Figure 1D), all of which have been validated to play critical roles in biotic or abiotic stresses. Additionally, we compared the protein sequences of *ZmDIR5* with these homologous genes and identified similar conserved amino acid regions (Figure 1E).

### 3.2. Expression Pattern Analysis and Subcellular Localization of ZmDIR5

**Figure 2:**
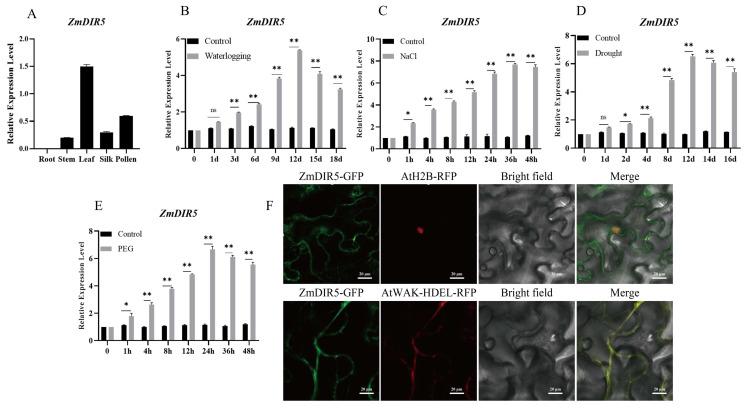
(**A**) Tissue expression pattern of ZmDIR5. (**B**–**E**) Induced expression patterns of ZmDIR5 under waterlogging, NaCl, drought, and PEG stress. Data are presented as the mean of triplicate values, with error represented as standard deviation (SD). Statistical significance is indicated as non-significant (ns), *p* < 0.05 (*), and *p* < 0.01 (**). (**F**) Subcellular localization of the ZmDIR5 protein. GFP fluorescence appears green, and RFP fluorescence appears red. The merged image displays an overlay of green and red fluorescence, resulting in yellow. Scale bar = 20 μm.

To further elucidate the expression pattern of *ZmDIR5*, we obtained its tissue expression profile from the RNA-seq database. Our analysis revealed that *ZmDIR5* is expressed in leaves, stems, silk, and pollen, with the highest expression levels observed in leaves and no detectable expression in roots (Figure 2A). Additionally, we conducted experiments that demonstrated *ZmDIR5* is upregulated to varying degrees in response to waterlogging (Figure 2B), salt (Figure 2C), drought (Figure 2D), and PEG (Figure 2E) treatments. Notably, the relative expression of *ZmDIR5* is significantly elevated under salt induction. To determine the subcellular localization of *ZmDIR5*, we performed colocalization experiments with the nuclear-localized protein AtH2B and the ER-localized protein AtWAK-HDEL. By utilizing the green fluorescence of GFP and the red fluorescence of RFP, which together emit yellow fluorescence, we observed the proteins using confocal microscopy. The results indicated that *ZmDIR5* colocalizes with AtWAK-HDEL in the endoplasmic reticulum (Figure 2F). The localization of *ZmDIR5* in the endoplasmic reticulum likely correlates with its role in the metabolic synthesis of lignans.

### 3.3. Mutation of ZmDIR5 Reduces the Ability of Maize Seedlings to Withstand Waterlogging Stress

**Figure 3:**
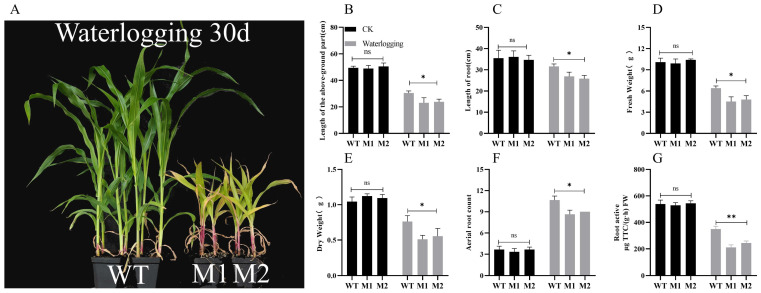
(**A**) Phenotype under waterlogging stress. (B–G) Phenotypic data of WT and mutants M1 and M2 under normal and waterlogging stress treatments over a period of 18 days. These data include measurements of plant height (**B**), root length (**C**), fresh weight (**D**), dry weight (**E**), number of adventitious roots (**F**), and root activity (**G**). Data are presented as the mean of triplicate values, with error represented as standard deviation (SD). Statistical significance is indicated as non-significant (ns), *p* < 0.05 (*), and *p* < 0.01 (**).

To further investigate the biological function of *ZmDIR5*, we subjected WT and mutant lines M1 and M2 to waterlogging treatment. After 30 days of waterlogging stress, we observed that the growth of WT plants was significantly stronger than that of the mutant lines M1 and M2 (Figure 3A). Additionally, we measured phenotypic data after 18 days of waterlogging treatment and found no significant differences in shoot length, root length, fresh and dry weight, number of adventitious roots, and root vigor between WT and mutant lines M1 and M2 under normal conditions. However, following waterlogging treatment, the shoot length of WT was significantly greater than that of the mutant lines (Figure 3B), with the WT’s root length being 17.5% and 22.5% longer than that of M1 and M2, respectively, indicating a significant difference (Figure 3C). Further measurements of plant fresh and dry weight after waterlogging stress revealed that the average fresh and dry weight of mutant lines M1 and M2 were significantly lower than those of the WT (Figure 3D,E), suggesting that the mutation of *ZmDIR5* adversely affected plant growth and development under waterlogging stress, as well as the accumulation of dry matter. During flooding or waterlogging stress, plants produce adventitious roots to cope with the stress and maintain root vigor, thereby enhancing oxygen contact and uptake [27]. After 18 days of waterlogging treatment, the number of adventitious roots in WT plants was 23% and 18.4% higher than that of mutants M1 and M2, respectively. Measurements of root vigor using a kit indicated that, under waterlogging stress, both the WT and mutant plants experienced a significant decrease in root vigor compared to normally grown plants; however, the decrease in root vigor in mutant lines M1 and M2 was significantly greater than that observed in WT. These results indicate that under waterlogging stress, the mutation of *ZmDIR5* leads to impaired root structure development and reduced root vigor, thereby causing more severe growth and development inhibition in the overall plant, suggesting that *ZmDIR5* positively regulates the waterlogging tolerance of maize seedlings.

### 3.4. ZmDIR5 Positively Regulates Salt Tolerance in Maize Seedlings

**Figure 4:**
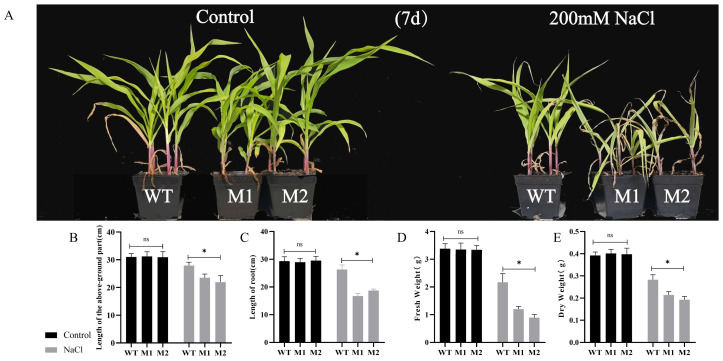
(**A**) Phenotypes observed under salt stress. (**B**–**E**) Phenotypic data for the WT and mutants M1 and M2 under both normal and salt stress treatments over a duration of 7 days, including measurements of plant height (**B**), root length (**C**), fresh weight (**D**), and dry weight (**E**). Data are presented as the mean of triplicate values, with error represented as standard deviation (SD). Statistical significance is indicated as non-significant (ns), *p* < 0.05 (*).

We conducted a salt tolerance experiment on maize seedlings during the three-leaf stage, exposing the WT and mutant lines M1 and M2 to both normal conditions and treatment with a 200 mM NaCl-Hogland solution. On the seventh day, no significant differences in growth were observed between the WT and the M1 and M2 mutant lines under normal conditions. However, under salt treatment, the growth inhibition in the M1 and M2 lines was significantly greater than that in the WT (Figure 4A). Under salt conditions, the plant height of the WT line was 18.7% and 27.4% greater than that of the M1 and M2 lines, respectively (Figure 4B). Additionally, the root length of the WT line was 57.5% and 40.6% longer than that of the M1 and M2 lines (Figure 4C). Furthermore, the average fresh weight (Figure 4D) and dry weight (Figure 4E) of the WT line were significantly higher than those of the mutant M1 and M2 lines.

### 3.5. ZmDIR5 Enhances Drought Tolerance in Maize Seedlings

**Figure 5:**
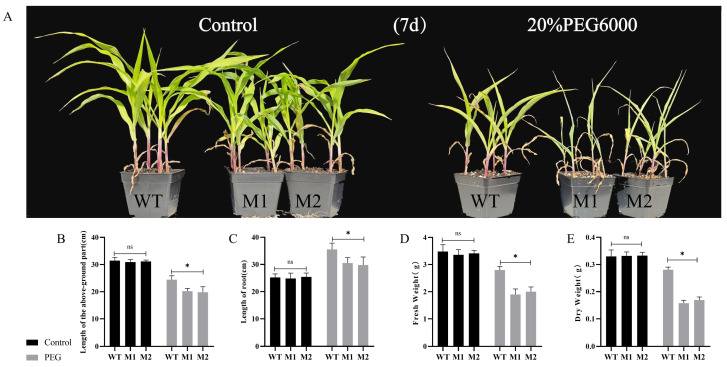
(**A**) Phenotypic appearance under drought stress. (**B**–**E**) Phenotypic data showing plant height (**B**), root length (**C**), fresh weight (**D**), and dry weight, (**E**) for WT and mutants M1 and M2, assessed under both normal and drought stress conditions after 7 days. Data are presented as the mean of triplicate values, with error represented as standard deviation (SD). Statistical significance is indicated as non-significant (ns), *p* < 0.05 (*).

We conducted a simulated drought experiment using a 20% PEG6000-Hogland solution on the WT and mutant lines M1 and M2. The results indicated that by the seventh day, wilting and chlorosis were more pronounced in the mutant lines compared to the wild type, suggesting inferior drought tolerance (Figure 5A). We subsequently measured growth indices, including plant height, root length, fresh weight, and dry weight. Under normal conditions, no significant differences were observed in plant height, root length, fresh weight, and dry weight between the WT and the M1 and M2 lines. However, under PEG treatment, the plant height, root length, fresh weight, and dry weight of WT plants (Figure 5B–E) were significantly greater than those of the M1 and M2 lines.

### 3.6. ZmDIR5 Enhances the Na^+^ and K^+^ Transport Capability of Plants Under Waterlogging, Salinity, and Drought Stress

**Figure 6:**
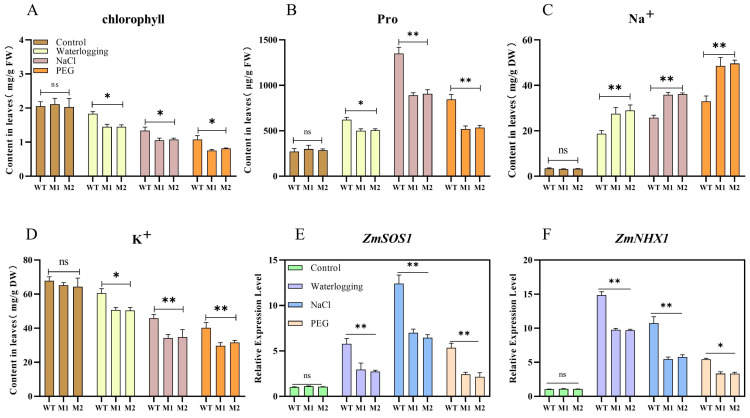
(**A**–**D**) Chlorophyll (**A**), proline (**B**), Na^+^ (**C**), and K^+^ (**D**) contents in the leaves of WT and mutant lines M1 and M2 after 7 days of exposure to three different stress treatments. (**E**,**F**) Relative expression analysis of ZmSOS1 (**E**) and ZmNHX1 (**F**) in the leaves of WT, M1, and M2 under the same stress conditions. Data are presented as the mean of triplicate values, with error represented as standard deviation (SD). Statistical significance is indicated as non-significant (ns), *p* < 0.05 (*), and *p* < 0.01 (**).

We conducted simultaneous experiments involving waterlogging stress, salinity stress, and drought stress. On the seventh day, the second leaves of maize seedlings from WT, M1, and M2 under different treatments were rapidly frozen in liquid nitrogen for subsequent analyses. We measured the chlorophyll content of maize seedlings subjected to various treatments and found that, under normal conditions, there was no significant difference in chlorophyll content between the leaves of WT and mutant lines. However, following treatment, the chlorophyll content in WT leaves was significantly higher than that in mutants M1 and M2 across all three stress conditions (Figure 6A), with the most pronounced chlorophyll loss occurring under drought stress. This observation suggests that the mutation in *ZmDIR5* exacerbates chlorophyll loss under the three stress conditions. Measurements of proline content in leaves indicated varying degrees of proline accumulation among WT, M1, and M2 plants under the three stress conditions. Notably, under waterlogging stress, the proline content in WT leaves was significantly higher than that in the mutant lines, while under salinity and drought stress, the proline content in WT leaves was markedly higher than in mutants M1 and M2 (Figure 6B).

We measured the sodium and potassium ion content in leaves and found that, under normal conditions, there was no significant difference in the sodium and potassium ion content between WT and mutant lines M1 and M2. However, under the three treatments, the accumulation of sodium ions in mutant plant leaves was significantly higher than in WT leaves (Figure 6C). Under waterlogging stress, potassium ion content in WT leaves was significantly higher than in the mutants, while under salinity and polyethylene glycol (PEG) treatments, potassium ion content in WT leaves was markedly higher than in mutants M1 and M2 (Figure 6D). *ZmSOS1* and *ZmNHX1* are two key genes associated with sodium and potassium ion transport, whose expression is induced by salinity and drought stress. Further analysis of the relative expression levels of these two important genes revealed that, under normal conditions, there was no significant difference in the relative expression levels of *ZmSOS1* and *ZmNHX1* between WT and mutant lines M1 and M2. However, under the three different treatments, the relative expression levels of *ZmSOS1* and *ZmNHX1* in WT leaves were significantly higher than those in mutant lines M1 and M2. Notably, under drought stress, the relative expression levels of *ZmSOS1* and *ZmNHX1* were the lowest (Figure 6E,F).

### 3.7. ZmDIR5 Enhances Antioxidant Capacity to Resist Three Types of Stress

**Figure 7:**
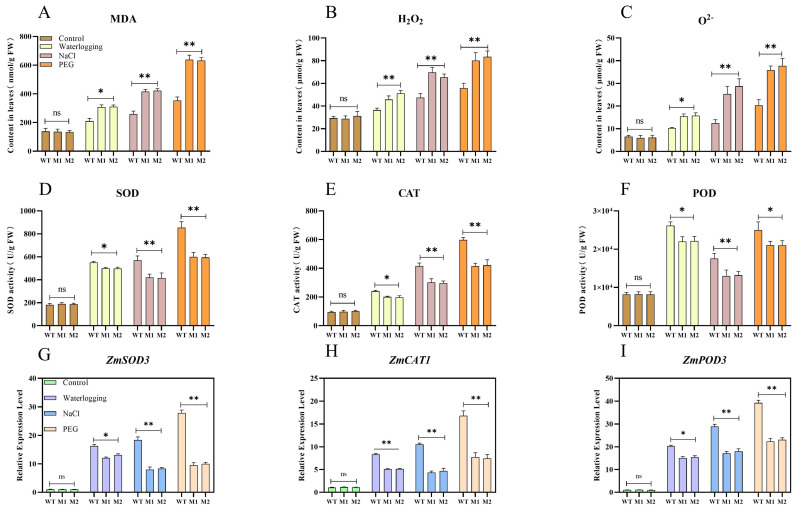
(**A**–**F**) MDA (**A**), H_2_O_2_ (**B**), and O^2−^ (**C**) contents, as well as SOD (**D**), CAT (**E**), and POD (**F**) enzyme activities in leaves of WT, M1, and M2 after 7 days of three stress treatments. (G–I) Relative expression analysis of *ZmSOD3* (**G**), *ZmCAT1* (**H**), and *ZmPOD3* (**I**) in leaves of WT, M1, and M2 under three stress treatments. Data are presented as the mean of triplicate values, with error represented as standard deviation (SD). Statistical significance is indicated as non-significant (ns), *p* < 0.05 (*), and *p* < 0.01 (**).

When plants encounter environmental stress, they frequently experience oxidative stress [28]. The rapid accumulation of ROS and MDA can disrupt cellular redox homeostasis and damage biomolecules, including cell membrane lipids, proteins, and nucleic acids, thereby fundamentally impairing the plant’s ability to withstand stress [29]. Consequently, we measured the accumulation of MDA, H_2_O_2_, and O^2−^ in plant leaves subjected to various treatments. Under normal conditions, the levels of these three oxidative stress-related metabolites in the leaves of WT and mutant lines M1 and M2 remained stable, with no significant differences observed. However, following the three treatments, the content of MDA, H_2_O_2_, and O^2−^ in the leaves of both WT and mutant lines increased rapidly, with the levels of these substances in WT leaves being significantly lower than those in mutant lines M1 and M2 across the different treatments (Figure 7A–C). Antioxidant enzymes play a crucial role in the defense mechanism of plant cells against oxidative stress, primarily including SOD, CAT and POD. These enzymes work synergistically to scavenge ROS and enhance the plant’s resilience to stress [30]. We assessed the activity of these three key antioxidant enzymes—SOD, CAT, and POD—in the leaves of different lines under various treatments. In contrast to the trends observed for the three oxidative-related metabolites, there were no significant differences in the activity of SOD, CAT, and POD enzymes in the leaves of WT, M1, and M2 lines under normal conditions. However, under the three stress treatments, the activity of the three enzymes in WT leaves was significantly higher than that in mutant lines M1 and M2. Notably, the activities of SOD and CAT were relatively higher in WT under salinity stress, while the activity of POD was highest in WT under waterlogging stress (Figure 7D–F). We further quantitatively analyzed the expression of key genes in the antioxidant enzyme defense pathway and found that the genes *ZmSOD3*, *ZmCAT1*, and *ZmPOD3* were significantly upregulated under all three stress treatments. Their relative expression levels in WT leaves were significantly higher than those in the mutant lines under salinity and drought stress. Under waterlogging stress, the relative expression levels of *ZmSOD3* and *ZmPOD3* in WT leaves were significantly higher than in the mutants, while the relative expression level of *ZmCAT1* was extremely significantly higher than in the mutant lines (Figure 7G–I).

### 3.8. Mutation of ZmDIR5 Reduces the Biosynthetic Capacity of ABA and Zeatin in Maize Seedlings

**Figure 8:**
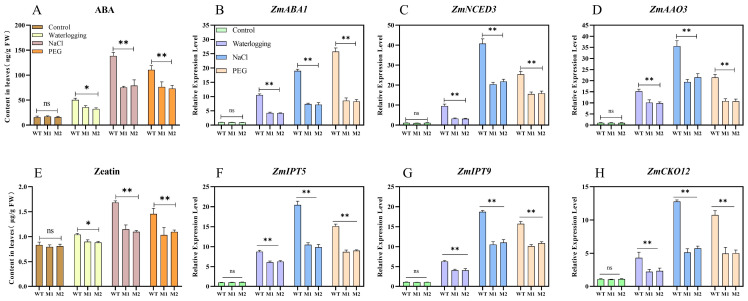
(**A**) ABA content in leaves of WT and mutants M1 and M2 after 7 days of three stress treatments. (**B**–**D**) Expression analysis of ABA biosynthesis-related genes in leaves under various treatments and genotypes. (**E**) Zeatin content in leaves of WT, M1, and M2 after 7 days of three stress treatments. (**F**–**H**) Expression analysis of zeatin biosynthesis-related genes in leaves under different treatments and genotypes. Data are presented as the mean of triplicate values, with error represented as standard deviation (SD). Statistical significance is indicated as non-significant (ns), *p* < 0.05 (*), and *p* < 0.01 (**).

ABA, a crucial plant hormone, plays a central role in the plant’s response to abiotic stresses. It helps maintain water balance during drought conditions by regulating stomatal closure, thereby reducing water loss through transpiration [31]. Additionally, ABA activates the expression of a series of stress-responsive genes, enhances the activity of antioxidant enzymes, and strengthens the plant’s antioxidant defense mechanisms, thus mitigating oxidative damage caused by stress [32]. Under salt stress, ABA aids in maintaining cellular ion balance by regulating the expression of ion channels and transport proteins [33]. Furthermore, in response to waterlogging and hypoxic conditions, plants synthesize ABA to transmit signals necessary for adaptation [34]. We measured the ABA content in plant leaves subjected to different treatments using GC–MS. Under normal conditions, there was no significant difference in ABA content between WT and mutant lines M1 and M2. However, under waterlogging treatment, the ABA content in the leaves of mutant lines M1 and M2 was significantly lower than that in WT, while under salt and PEG treatments, it was extremely significantly lower (Figure 8A). We subsequently conducted a quantitative analysis of several key genes involved in ABA biosynthesis (*ZmABA1*/*ZmNCED3*/*ZmAAO3*) [35]. Under normal conditions, the relative expression levels of *ZmABA1*, *ZmNCED3* and *ZmAAO3* did not differ significantly among the lines. However, under the three stress treatments, these genes were significantly upregulated, and their relative expression levels in WT leaves were extremely significantly higher than in mutant lines M1 and M2. Notably, the relative expression levels of *ZmNCED3* and *ZmAAO3* were highest in WT leaves under salt treatment, while *ZmABA1* was highest in WT leaves under PEG treatment (Figure 8B–D).

Zeatin, a natural cytokinin, plays a crucial role not only in plant growth and development but also in enhancing plant resistance to abiotic stresses through various mechanisms [36]. We continued to measure zeatin content in maize leaves using LC–MS. The results indicated that under normal conditions, there was no significant difference in zeatin content among the lines. However, under salt and PEG treatments, the zeatin content in WT leaves was significantly higher than in mutant lines M1 and M2, and only under waterlogging treatment was it significantly elevated (Figure 8E). *ZmIPT5*, *ZmIPT9*, and *ZmCKO12* are critical enzyme synthesis genes in the AMP synthesis pathway of zeatin [37,38]. Further analysis of the relative expression levels of these genes revealed a trend consistent with zeatin synthesis. Under the three stress treatments, the relative expression levels of *ZmIPT5*, *ZmIPT9* and *ZmCKO12* in the leaves of mutant lines M1 and M2 were significantly lower than those in WT, with the highest expression observed under salt treatment and the lowest under waterlogging treatment (Figure 8F–H).

### 3.9. ZmDIR5 Mutation Affects Total Lignan Synthesis in Maize Seedlings Under Stress

**Figure 9:**
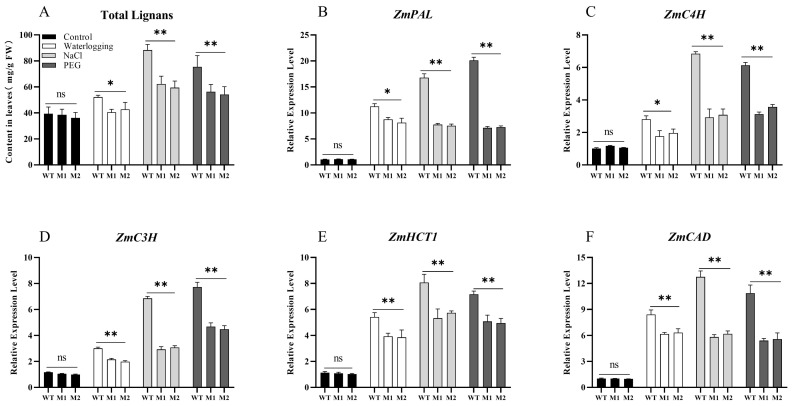
(**A**) Total lignan content in the leaves of WT, M1, and M2 after 7 days of three stress treatments. (**B**–**F**) Expression analysis of genes associated with the phenylpropanoid biosynthesis pathway in leaves subjected to different treatments and genotypes. Data are presented as the mean of triplicate values, with error represented as standard deviation (SD). Statistical significance is indicated as non-significant (ns), *p* < 0.05 (*), and *p* < 0.01 (**).

Lignans are natural compounds formed by the polymerization of two phenylpropanoid derivatives, specifically C6-C3 monomers. They typically exist in a free state, with some forming glycosides with sugars, and exhibit various biological activities, including antioxidant properties. Total lignans represent a mixture of multiple types of these compounds. Dirigent proteins play a crucial role in lignan synthesis within the phenylpropanoid biosynthetic pathway, facilitating the formation of the lignan monomer pinoresinol from coniferyl alcohol through stereospecific coupling, an essential step for successful lignan synthesis [39]. We measured the total lignan content in the leaves of maize seedlings subjected to different treatments using the solid-phase sandwich method. The results indicated that, under normal conditions, the total lignan content in mutant lines was slightly lower than that in WT plants, although the difference was not statistically significant. Following various stress treatments, the total lignan content in the leaves of both WT and mutant lines increased to some extent, with the total lignan content in WT leaves being significantly higher than in mutant lines M1 and M2 under stress conditions (Figure 9A). Previous studies have indicated that under water deficit stress, key enzyme genes in the lignan synthesis pathway, such as *ZmC3H* and *ZmC4H*, are upregulated in maize leaves [40]. Building on this, we retrieved homologous genes related to lignan synthesis from the MaizeGDB and NCBI databases, including *ZmPAL*, *ZmC3H*, *ZmC4H*, *ZmHCT1*, and *ZmCAD* [41], and quantitatively analyzed their expression levels under various treatments. The results demonstrated that under normal conditions, there were no significant differences in the relative expression levels of these genes between the WT and mutant lines. However, following the three stress treatments, all genes were upregulated. Notably, under waterlogging treatment, the relative expression levels of *ZmPAL* and *ZmC4H* in WT leaves were significantly higher than those in mutant lines M1 and M2, while *ZmC3H*, *ZmHCT1*, and *ZmCAD* exhibited extremely significant increases compared to mutant lines M1 and M2. Additionally, the relative expression levels of these five genes in WT leaves under salinity and PEG treatments were also extremely significantly higher than in mutant lines (Figure 9B–F).

## 4. Discussion

*DIR* genes are present in all terrestrial plants but are absent in aquatic plants, marking a significant evolutionary transition from aquatic to terrestrial environments. These genes play crucial roles in plant growth and development, as well as in responses to both biotic and abiotic stresses. Furthermore, *DIR* genes are essential for lignan synthesis, and their functions were first elucidated by Davin in Forsythia suspensa [42]; they are now widely studied in the context of traditional Chinese medicine and various crops. Currently, research on *DIR* genes in maize is limited, with only *ZmESBL* previously identified as being associated with the development of the Casparian strip in maize and enhancing its salt tolerance [43]. However, in this study, no correlation was observed between *ZmDIR5* and the development of the Casparian strip. In this study, bioinformatics analysis revealed a high degree of homology between this gene and several crop *DIR* genes that have been validated for their roles in disease resistance and tolerance to drought and salinity (Figure 2D). The protein sequences also exhibit numerous conserved amino acid regions (Figure 2E). The promoter region contains common abiotic stress-related elements, such as MYB, MYC, ARE, DRE-core, and STRE (Figure 2C), suggesting potential functions in response to abiotic stresses. Subcellular localization studies revealed that the ZmDIR5 protein is situated in the endoplasmic reticulum (Figure 3F), which may be associated with its biological function. We induced the expression of this gene under various abiotic stresses and observed that it is significantly upregulated only in response to waterlogging, salinity, drought and PEG stresses (Figure 3B–E). Consequently, we constructed EMS mutant lines of *ZmDIR5* and performed stress treatment experiments. Experimental validation revealed that under conditions of waterlogging, salinity, and drought stress, the growth of *ZmDIR5* mutant lines was significantly impaired compared to that of WT plants. After 30 days of waterlogging stress, the mutant maize plants exhibited a notable reduction in height compared to the WT, accompanied by more pronounced leaf yellowing (Figure 3A). Similarly, under salt stress and PEG-simulated drought conditions, the wilting and chlorosis observed in the mutant lines were more severe than those in the WT (Figure 4A and Figure 5A). These findings indicate that the mutation of ZmDIR5 diminishes the resistance of maize seedlings to waterlogging, salinity, and drought stress, thereby hindering the growth and dry matter accumulation of the mutant plants. Furthermore, we assessed the chlorophyll content in the leaves of both WT and mutant plants under the three stress conditions. The results demonstrated that the chlorophyll content in WT was significantly higher than that in the mutants following stress treatment (Figure 6A), which aligns with the phenotypic observations. This suggests that *ZmDIR5* plays a role in mitigating stress by inhibiting chlorophyll degradation. Proline is crucial for plant resistance to abiotic stress, as it helps maintain cellular osmotic pressure, thereby mitigating damage to plant cells under adverse conditions. It is recognized as an important stress-related metabolite [44]. This aligns with our findings that the Pro content in the leaves of mutant plants under the three stress conditions was significantly lower than that in the WT (Figure 6B), indicating that the mutation of *ZmDIR5* also reduces the plant’s Pro metabolic capacity, consequently diminishing the osmotic regulation capacity of plant cells. The balance of Na^+^ and K^+^ is essential for maintaining cellular osmotic equilibrium and preventing chlorophyll loss [45]. *ZmSOS1* and *ZmNHX1* are known to be expressed under stress conditions to regulate Na^+^ and K^+^ levels, thereby maintaining ion balance and enhancing plant adaptability to adverse conditions [46,47]. Under the three stress conditions, the Na^+^ content in the leaves of mutant plants was significantly higher than that in the WT, while the K^+^ content was significantly lower. Additionally, the expression of the *ZmSOS1* and *ZmNHX1* genes was significantly lower in the mutants compared to the WT. This indicates that *ZmDIR5* can reduce Na^+^ accumulation and K^+^ loss in maize plants under waterlogging, salt, and drought stress, while enhancing the expression of *ZmSOS1* and *ZmNHX1* genes. This alleviates ion toxicity and osmotic stress, thereby improving plant stress tolerance. These results are presented in Figure 6C–F. The accumulation of MDA and ROS exacerbates cellular damage and diminishes plant stress tolerance. Conversely, the antioxidant enzyme defense system enhances the antioxidant capacity and stress resistance of plants. Our findings indicate that under three different stress conditions, the accumulation of MDA, H_2_O_2_, and O_2_^−^ in mutant plants was significantly greater than that in WT plants (Figure 7A–C). Additionally, the activities of SOD, CAT, and POD were markedly lower in the mutants (Figure 7D–F). Furthermore, the expression levels of *ZmSOD3*, *ZmCAT1* and *ZmPOD3*, which are key genes associated with the antioxidant defense system, were significantly reduced in the mutant plants under all three stress conditions (Figure 7G–I). These results suggest that *ZmDIR5* enhances the antioxidant capacity of maize plants by upregulating the expression of relevant genes and increasing the activity of antioxidant enzymes, thereby mitigating the accumulation of ROS and MDA. ABA and zeatin are critical hormones in the plant stress response system. We measured the contents of ABA and zeatin, along with the expression levels of associated genes, in both WT and mutant plants under various stress conditions. The results demonstrated that the ABA content and the expression levels of key genes in the ABA signaling pathway, including *ZmABA1*, *ZmNCED3* and *ZmAAO3*, were significantly higher in the WT compared to the mutants (Figure 8A–D). Similarly, the zeatin content and the expression levels of key genes involved in zeatin synthesis, such as *ZmIPT5*, *ZmIPT9*, and *ZmCKO12*, exhibited the same trend (Figure 8E–H). These findings indicate that *ZmDIR5* can affect the expression of these critical genes and enhance the synthesis of ABA and zeatin under waterlogging, salt, and drought stress, thereby improving plant stress tolerance through the accumulation of these essential hormones. Our findings also indicate that *ZmDIR5* significantly influences the accumulation of total lignans in maize leaves and the expression of genes associated with the phenylpropanoid biosynthesis pathway, as illustrated in Figure 9A–F. Under conditions of waterlogging, salinity, and drought stress, the total lignan content in the leaves of mutant plants was markedly lower than that in the WT (Figure 9A), suggesting that the mutation of *ZmDIR5* impairs lignan synthesis. Previous studies have demonstrated that, under water-deficient conditions, genes involved in the phenylpropanoid biosynthesis pathway, such as *ZmC3H* and *ZmC4H*, are significantly upregulated in maize. We assessed the relative expression levels of *ZmC3H*, *ZmC4H*, and other genes within the phenylpropanoid biosynthesis pathway, including *ZmPAL*, *ZmHCT1*, and *ZmCAD*, across the three stress conditions. The results indicated that these genes were upregulated under stress; however, their expression levels in the mutants were significantly lower compared to the WT (Figure 9B–F). This observation suggests that the mutation of *ZmDIR5* suppresses the expression of these genes under three stress conditions, thereby reducing the overall accumulation of lignans under stress. Furthermore, this implies that *ZmDIR5* likely enhances plant stress tolerance by promoting lignan synthesis, potentially linked to the strong antioxidant capacity of lignans, as reported in previous studies. In conclusion, *ZmDIR5* is a stress-responsive gene that positively regulates the tolerance of maize seedlings to waterlogging, salinity, and drought stress.

Previous studies have identified several genes, akin to investigated in this research, that endow maize with the ability to modulate tolerance to various abiotic stresses. For instance, *ZmWRKY106*, a member of the WRKY transcription factor family, has been shown to significantly enhance Arabidopsis tolerance to high temperatures and drought [48]. The bZIP transcription factor family member *ZmbZIP*4, when overexpressed, increases the number of lateral roots, elongates primary roots, and enhances the overall root system, thereby improving maize resistance to high temperatures, low temperatures, salinity, and drought [49]. Similarly, the overexpression of *ZmSRG7* (a stress-related gene) enhances maize’s tolerance to drought and salt [50]. However, no studies have demonstrated that a single gene can simultaneously confer resistance to waterlogging, salinity, and drought stresses, as observed with *ZmDIR5*. Plants respond to abiotic stresses through a series of complex physiological and biochemical mechanisms, which include ion balance, osmotic regulation, oxidative defense systems, and the synthesis and signal transduction of plant hormones, all of which coordinate plant growth and stress responses. Throughout our experiments, we conducted analyses focusing on the aforementioned aspects and discovered that *ZmDIR5* significantly enhances the tolerance of maize plants to waterlogging, salinity, and drought stress by influencing multiple pathways, including the phenylpropanoid biosynthesis pathway. Furthermore, the primary mechanisms by which *ZmDIR5* responds to adverse conditions may vary under different stress scenarios. Our research indicates that, under waterlogging stress, *ZmDIR5* may primarily promote the formation of aerenchyma in maize roots and enhance the roots’ oxygen acquisition capabilities, as well as increase the expression of ethylene synthesis-related genes (Figure S3) to improve waterlogging tolerance. In the context of salinity stress, *ZmDIR5* may concentrate on strengthening ion balance and mechanisms related to plant hormone synthesis and signal transduction, thereby enhancing salt tolerance in maize seedlings. Under drought stress, *ZmDIR5* may activate antioxidant defense mechanisms to bolster maize drought tolerance. The *ZmDIR5* gene holds significant potential for improving maize resilience to waterlogging, salinity, and drought. These functions position *ZmDIR5* as a promising target for future maize stress breeding initiatives, aimed at developing new maize varieties with enhanced stress adaptation capabilities, ultimately contributing to the stability and sustainability of agricultural production.

Throughout the experiment, data from waterlogging stress were significantly lower than those from salinity and drought stresses in terms of physiological metabolism, enzyme activity, and the synthesis of hormones such as ABA and zeatin, as well as in the regulation of related gene expression. This discrepancy may be attributed to the nature of waterlogging stress, which is a long-term and relatively chronic abiotic stress. The hypoxia it induces is less severe compared to that caused by salinity and drought stresses; consequently, the overall data were significantly lower than those of the other two treatments by the seventh day. Nevertheless, we also measured relevant metabolic indicators and enzyme activities for materials subjected to waterlogging up to the 18th day, where we observed a sustained upregulation of metabolic indicators, indicating that the stress on the mutant was intensified, as illustrated in Figure S2. Typically, plants encounter complex environmental stresses rather than isolated stressors. For instance, combined stresses such as drought and salinity, waterlogging and salinity, or drought and high temperature tend to be more destructive when they occur concurrently [51]. Therefore, the stability of *ZmDIR5* under combined stresses warrants further investigation.

In this study, we observed that *ZmDIR5* influences the expression of multiple genes across various pathways. However, it remains unclear whether these effects arise from ZmDIR5 individually regulating each gene in a one-to-one manner or if *ZmDIR5* preferentially modulates the expression of specific genes, thereby initiating a hierarchical regulatory cascade that leads to comprehensive changes. This aspect has not been thoroughly validated in the current research.

The mutation in *ZmDIR5* also influenced the total lignan content in maize leaves under both normal and stress conditions (Figure 9A). This effect may arise from *ZmDIR5’*s involvement in the synthesis of one or more types of lignans that do not significantly contribute to normal growth and development, resulting in a slightly lower total lignan content in mutants compared to the WT under normal conditions. Under environmental stress, the mutation in *ZmDIR5* hinders the rapid synthesis and accumulation of these lignans, leading to marked differences from the WT following treatment. These lignans may confer stress resistance through their antioxidant activity. However, we did not detect the target lignan components via metabolomic analysis, potentially due to the limited research on lignans in maize and the absence of detailed studies on the types and quantities of lignans present. Consequently, our future research will aim to identify all lignan types in maize leaves, ascertain the target lignans associated with *ZmDIR5*, and complete the biological function analysis of *ZmDIR5*, thereby providing a new metabolic indicator for maize stress resistance.

In conclusion, this study demonstrates that the mutant plants exhibited reduced resistance to waterlogging, salinity, and drought stresses when compared to the wild type. This phenomenon may be attributed to the disruption of various physiological and biochemical mechanisms, including internal ion balance, osmotic regulation, antioxidant defense systems, and the synthesis and signal transduction of ABA and zeatin, which are affected by the mutation of *ZmDIR5*. The expression of *ZmDIR5* has the potential to enhance plant resistance to waterlogging, salinity, and drought by fortifying these mechanisms, thereby acting as a positive regulator for these three types of stress. Our findings may provide a theoretical basis and genetic resources for the breeding of maize varieties with enhanced stress adaptation capabilities.

## 5. Conclusions

In summary, this study identifies *ZmDIR5* as a pivotal regulator of maize tolerance to waterlogging, salinity, and drought stresses. Functional characterization of ZmDIR5 mutants revealed compromised stress resilience, marked by impaired physiological and biochemical responses, including disrupted ion homeostasis, reduced osmotic regulation, diminished antioxidant enzyme activity, and altered synthesis of ABA and zeatin. ZmDIR5, localized to the endoplasmic reticulum, likely facilitates lignan biosynthesis, with its mutation leading to decreased total lignans under stress. Notably, *ZmDIR5* represents an example of a single gene conferring resistance to multiple abiotic stresses, distinguishing it from previously reported stress-specific regulators. While its mechanisms vary across stress types (e.g., aerenchyma formation in waterlogging, antioxidant activation in drought), ZmDIR5 consistently acts as a positive modulator, integrating stress signaling and metabolic pathways. However, the specific lignan species linked to *ZmDIR5* and its performance under combined stresses remain unresolved. This work not only advances our understanding of *DIR* gene functionality in abiotic stress adaptation but also positions *ZmDIR5* as a promising candidate for breeding multi-stress-resilient maize varieties. Future studies should focus on elucidating ZmDIR5-associated lignan diversity, protein interaction networks, and its efficacy in complex field conditions to unlock its full agronomic potential.

## Data Availability

The original contributions presented in this study are included in the article/Supplementary Materials. Further inquiries can be directed to the corresponding authors.

## Supplementary Materials

The following supporting information can be downloaded at: https://www.mdpi.com/article/10.3390/plants14050785/s1, Figure S1: The mutation situation of ZmDIR5; Figure S2: Waterlogging treatment for 18 days, WT and mutant related index data; Figure S3: Relative expression analysis of ethylene synthesis-related genes ZmACS2, ZmACS6, and ZmACS7 in leaves of WT and M1, M2 after 7days of three stress treatments; Table S1: ZmDIR5-EMS mutant gene mutation details table; Table S2: Primers and their sequences used in this study.
