# Supplementary material for: Mutation of ZmDIR5 Reduces Maize Tolerance to Waterlogging, Salinity, and Drought
Source: Plants (Basel). 2025 Mar 4;14(5):785. doi: 10.3390/plants14050785 (PMC11902002; doi:10.3390/plants14050785)
Supplement: Supplementary file 1 [file plants-14-00785-s001.zip › Supplementary Table1.pdf]

Supplementary Table S1

ZmDIR5-EMS Mutant Gene Mutation Details Table

GeneID	Chr	Loc	Ref	Mut	Effect	Codon_Change	AA_Change	MutantID
Zm00001d006873	2	218317107	C	T	STOP_GAINED	Cag/Tag	Q/*	EMS4-03f631
	2	218316955	G	A	NON_SYNONYMOUS_CODI...	cGc/cAc	R/H	EMS4-1ee828
