# Supplementary material for: Mutation of ZmDIR5 Reduces Maize Tolerance to Waterlogging, Salinity, and Drought
Source: Plants (Basel). 2025 Mar 4;14(5):785. doi: 10.3390/plants14050785 (PMC11902002; doi:10.3390/plants14050785)
Supplement: Supplementary file 1 [file plants-14-00785-s001.zip › Supplementary Table2.pdf]

Supplementary Table S2

Various primers and their sequences used in the paper

Prime	GeneID	Primer sequence
ZmDIR5-F	Zm00001d006873	5' ATGCCCATTGGCCATTACAACC 3'
ZmDIR5-R	Zm00001d006873	5' CGACCTGGAGCAGCGAGAACCC 3'
ZmDIR5-RT-qPCR-F	Zm00001d006873	5' CTACTTCTCGGCCATCAC 3'
ZmDIR5-RT-qPCR-R	Zm00001d006873	5' AAGACGACGAGGTCAAGC 3'
ZmACTINE1-RT-qPCR-F	Zm00001eb216070	5' ATGTTTCCTCCCATTGCCGAT 3'
ZmACTINE1-RT-qPCR-R	Zm00001eb216070	5' CCAGTTTCGTCATACTCTCCCTTG 3'
ZmCAT1-RT-qPCR-F	Zm00001eb228570	5' GGAACAACAACTCTGCCCTCACCG 3'
ZmCAT1-RT-qPCR-R	Zm00001eb228570	5' CAAAGAAACCCTTGGCACTGGCTC 3'
ZmSOD3--RT-qPCR-F	Zm00001eb285090	5' ATCAAGTTCAACGGCGGTGGTCAT 3'
ZmSOD3--RT-qPCR-R	Zm00001eb285090	5' GCTAACCACACCCATCCAGACCCT 3'
ZmPOD3-RT-qPCR-F	Zm00001eb330540	5' TCATAACAGCGTTCGGCAACAAGG 3'
ZmPOD3-RT-qPCR-R	Zm00001eb330540	5' GTCGTTGTAGATGTGGTCCCTGAA 3'
ZmABA1-RT-qPCR-F	Zm00001eb251960	5' TTCCAGCAGGGCGACTACC 3'
ZmABA1-RT-qPCR-R	Zm00001eb251960	5' CCTTCTCCAGGGTCAGGTAGCACT 3'
ZmNCED3-RT-qPCR-F	Zm00001eb181940	5' ATGTCGTGTCCAAGCCGTTC 3'
ZmNCED3-RT-qPCR-R	Zm00001eb181940	5' AGCACCATCTCCTGGAGCTTGAA 3'
ZmAAO3-RT-qPCR-F	Zm00001eb305060	5' GCAAGGTGCTTAGGTATTCCATTC 3'
ZmAAO3-RT-qPCR-R	Zm00001eb305060	5' CCCATCGTTCTTGAATCCGACAGT 3'
ZmIPT5-RT-qPCR-R	Zm00001eb212350	5' GTGCTGCTTCCTCTGGGTGG 3'
ZmIPT5-RT-qPCR-F	Zm00001eb212350	5' GGGACGTGTTGGCCTTGATC 3'
ZmIPT9-RT-qPCR-F	Zm00001eb095250	5' CACGAACAAGGTGACGGA 3'
ZmIPT9-RT-qPCR-R	Zm00001eb095250	5' CCCAGAGGAAGCAGCAGT 3'
ZmCKO12-RT-qPCR-F	Zm00001eb076990	5' AGCCCGGAGCTTTTCTTC 3'
ZmCKO12-RT-qPCR-R	Zm00001eb076990	5' CTTGTTGGTGCCGTCGTC 3'
ZmACS2-RT-qPCR-F	Zm00001eb073290	5' TGCTGGAGGTCAGGCGTG 3'
ZmASC2-RT-qPCR-R	Zm00001eb073290	5' GGGTTGGAGGGGTTGGTG 3'
ZmASC6-RT-qPCR-F	Zm00001eb055950	5' GCTCATCACCAACCCTTC 3'
ZmASC6-RT-qPCR-R	Zm00001eb055950	5' ACCGTGCCCGAGTATATC 3'
ZmASC7-RT-qPCR-F	Zm00001eb428490	5' TCTCGTGGATGGACCTGC 3'
ZmASC7-RT-qPCR-R	Zm00001eb428490	5' CGGCCTTGGCCTTGCTGT 3'
ZmSOS1-RT-qPCR-F	Zm00001eb033240	5' TCATCATCCTCACAATGGCTCTAA 3'
ZmSOS1-RT-qPCR-R	Zm00001eb033240	5' ACCAACTTGCGTGGGACAACTTTA 3'
ZmNHX1-RT-qPCR-F	Zm00001eb165870	5' TAGAGAAGTGGAAGATTGTCGG 3'
ZmNHX1-RT-qPCR-R	Zm00001eb165870	5' CTAGGTTGTTGAGTATGGCCTG 3'
ZmC4H-RT-qPCR-F	Zm00001eb368900	5' GACAGCAACGGGCTCAAGTG 3'
ZmC4H-RT-qPCR-R	Zm00001eb368900	5' AGGTAGGGGAGGTTGTGCGT 3'
Zm4CL-RT-qPCR-F	Zm00001eb233720	5' TGCTGCCGCTGTTCCACATCTA 3'

Zm4CL-RT-qPCR-R	Zm00001eb233720	5'	CGAGCACGGCATTGGGAATCTT	3'
ZmC3H-RT-qPCR-F	Zm00001eb291460	5'	TTCAACAACATAACAAGGCTGGCA	3'
ZmC3H-RT-qPCR-R	Zm00001eb291460	5'	TCATTGTCAGGCGGTCCCTTCTCT	
ZmCAD-RT-qPCR-F	Zm00001eb071040	5'	TCAGCGGTGATGCTAACCAGATG	3'
ZmCAD-RT-qPCR-R	Zm00001eb071040	5'	GCCCCCAGTAACCAAAGAGAAGG	3'
ZmPAL-RT-qPCR-F	Zm00001eb185250	5'	CCGCTGTACCGGTTCGTG	3'
ZmPAL-RT-qPCR-R	Zm00001eb185250	5'	TGCTTGCGCTGGTTGATG	3'
ZmHCT1-RT-qPCR-F	Zm00001eb006410	5'	GCAGCCCGTCTACTCCAC	3'
ZmHCT1-RT-qPCR-R	Zm00001eb006410	5'	GACAGGTCAGCCGAGATT	3'
