# Supplementary figures and images for: Mutation of ZmDIR5 Reduces Maize Tolerance to Waterlogging, Salinity, and Drought
Source: Plants (Basel). 2025 Mar 4;14(5):785. doi: 10.3390/plants14050785 (PMC11902002; doi:10.3390/plants14050785)

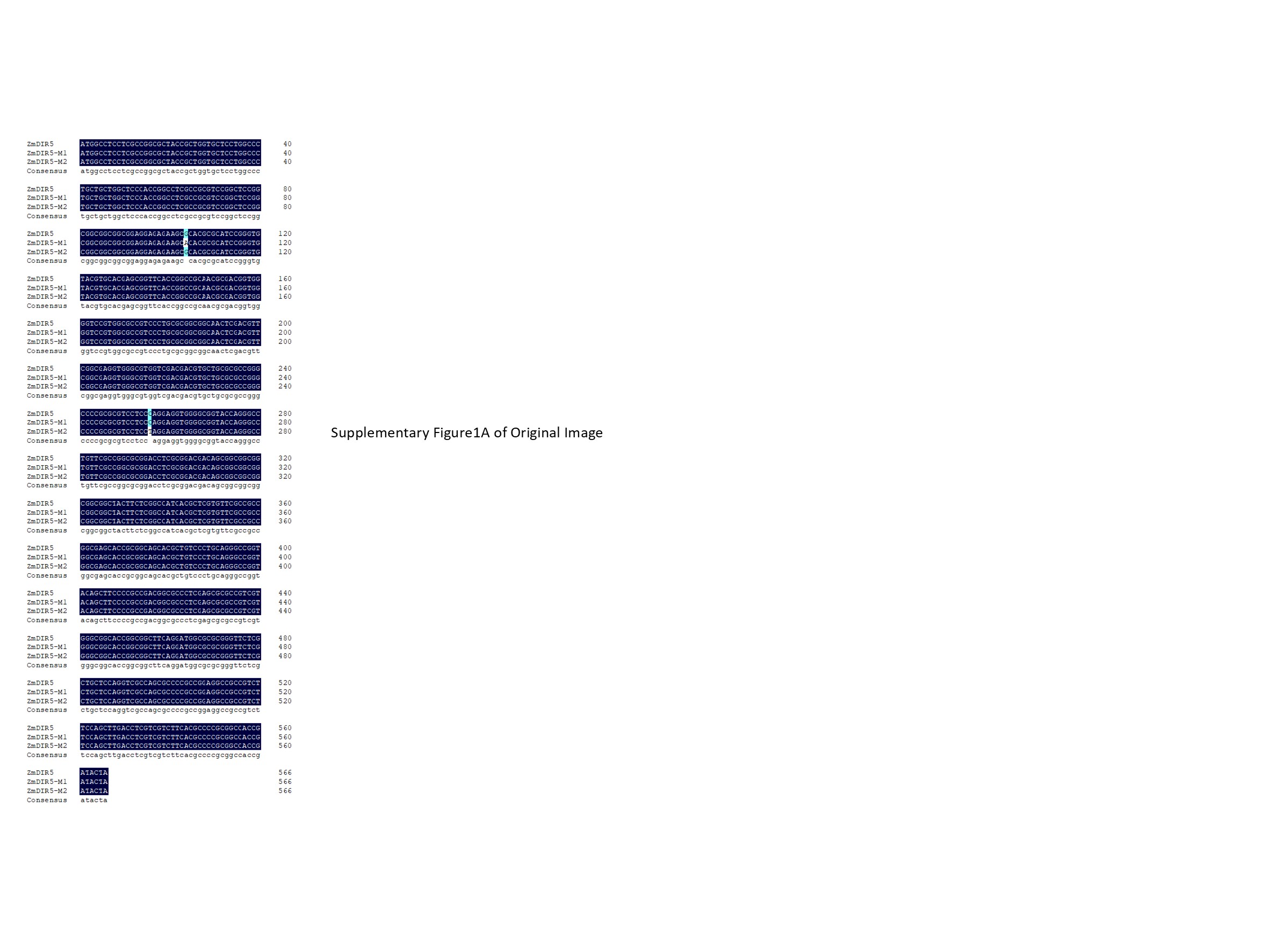

Supplement: Supplementary file 1 [file plants-14-00785-s001.zip › Supplementary Figure1-2.JPG]

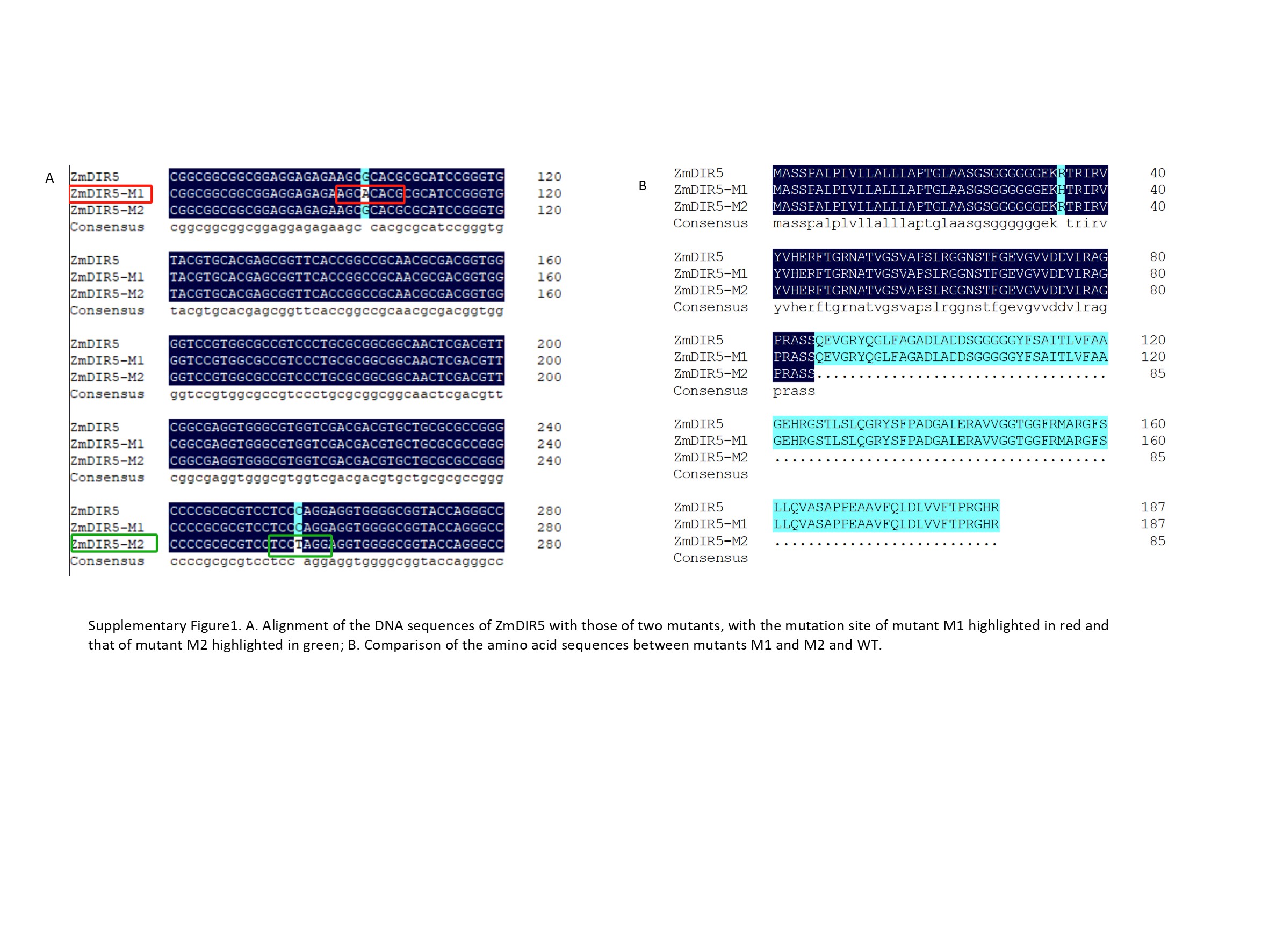

Supplement: Supplementary file 1 [file plants-14-00785-s001.zip › Supplementary Figure1.JPG]

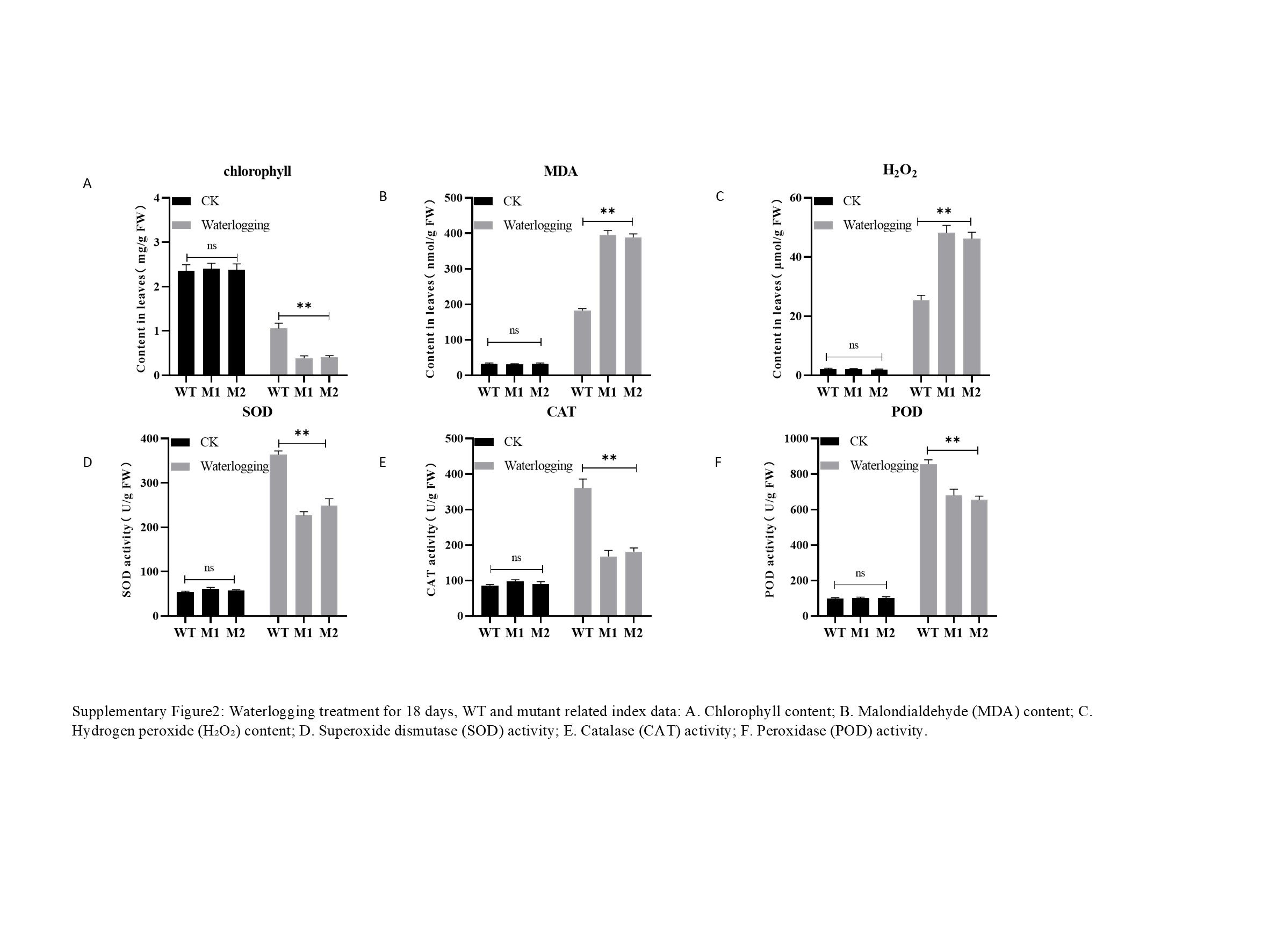

Supplement: Supplementary file 1 [file plants-14-00785-s001.zip › Supplementary Figure2.JPG]

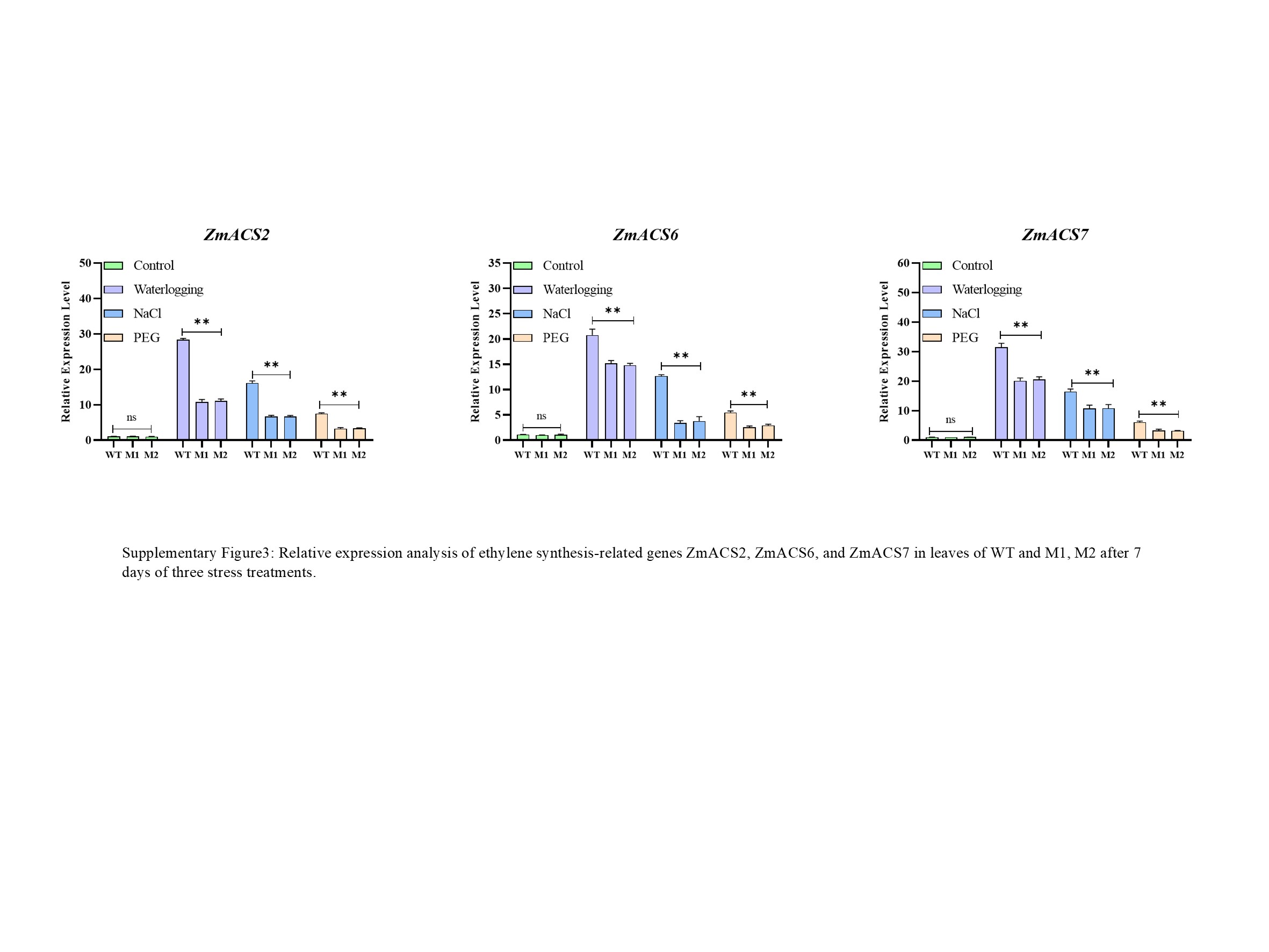

Supplement: Supplementary file 1 [file plants-14-00785-s001.zip › Supplementary Figure3.JPG]
